# Recognition of adverse drug events in older hospitalized medical patients

**DOI:** 10.1007/s00228-012-1316-4

**Published:** 2012-06-07

**Authors:** Joanna E. Klopotowska, Peter C. Wierenga, Susanne M. Smorenburg, Clementine C. M. Stuijt, Lambertus Arisz, Paul F. M. Kuks, Marcel G. W. Dijkgraaf, Loraine Lie-A-Huen, Sophia E. de Rooij, on behalf of the WINGS study group

**Affiliations:** 1 Department of Hospital Pharmacy, Academic Medical Centre, Meibergdreef 9, 1105 AZ Amsterdam, The Netherlands; 2 Department of Hospital Pharmacy, Deventer Hospital, Nico Bolkesteinlaan 75, 7416 SE Deventer, The Netherlands; 3 Section of Geriatrics, Department of Internal Medicine, Academic Medical Centre, Meibergdreef 9, 1105 AZ Amsterdam, The Netherlands; 4 Department of Internal Medicine, Academic Medical Centre, Meibergdreef 9, 1105 AZ Amsterdam, The Netherlands; 5 Clinical Research Unit, Academic Medical Centre, Meibergdreef 9, 1105 AZ Amsterdam, The Netherlands

**Keywords:** Adverse drug events, Medication safety, Elderly, Hospital

## Abstract

**Objective:**

To assess medical teams’ ability to recognize adverse drug events (ADEs) in older inpatients.

**Methods:**

The study cohort comprised 250 patients aged 65 years or older consecutively admitted to Internal Medicine wards of three hospitals in the Netherlands between April and November 2007. An independent expert team identified ADEs present upon admission or occurring during hospitalization by a structured retrospective patient chart review. For all ADEs identified, the expert team assessed causality, severity, preventability, and recognition by medical teams.

**Results:**

The medical teams did not recognize 19.9 % of all ADEs present upon admission {60.4 ADEs [95 % confidence interval (CI) 51.5–70.8] per 100 hospitalizations} and 20.3 % of all ADEs occurring during the hospital stay [47.2 ADEs (95 % CI 39.4–56.5) per 100 hospitalizations]. Unrecognized ADEs were significantly more often ADEs with possible causality (p=0.014, df=1), ADEs caused by medication errors (p<0.001, df=1), and ADEs not manifesting as new symptoms (p<0.001, df=1). The medical teams did not recognize 23.2 % of mild to moderately severe ADEs and 16.5 % of severe, life-threatening, or fatal ADEs. The recognition of ADEs varied with event type.

**Conclusions:**

The recognition of ADEs by medical teams was substantial for those ADEs with evident causality and with clinically apparent and severe consequences. ADEs mimicking underlying pathologies with a lower severity went unrecognized much more often, as did those resulting only in abnormal laboratory values. Tools to improve the recognition of ADEs by medical teams should, therefore, focus on those ADEs that are more challenging to detect.

## Introduction

Adverse Drug Events (ADEs) are the most frequent type of adverse events in medical inpatients [1] and are associated with a prolonged hospital stay, a twofold increase in the risk of death, and higher costs [2]. Approximately 50 % of ADEs are preventable [3]. A widely accepted definition of an ADE is any harm occurring during drug therapy which may result from either appropriate care (non-preventable ADE) or from suboptimal care (preventable ADE) (i.e., a medication error) [4].

It is widely acknowledged that older patients are especially at risk for ADEs [5, 6], primarily due to multiple co-morbidities, polypharmacy, and higher vulnerability due to decreased organ function, increased susceptibility to drugs, and frequently present cognitive impairment [7, 8]. Furthermore, correct and timely diagnosis is often hampered by atypical disease presentation, such as falls or delirium [5, 9]. Therefore, not only avoiding ADEs in older inpatients, but also, when they do occur, timely recognition of ADEs may pose a significant challenge for medical teams [9, 10]. Failure to recognize ADEs during the hospital stay may lead to inappropriate actions causing even more harm [10]. The study by Nebeker et al. [11] reports that in a general inpatient population, 24 % of ADEs subsequently identified by the researchers were not recognized by the medical teams.

Although the body of literature on the occurrence of (preventable) ADEs in older hospitalized patients is extensive [5, 12–22], data on medical teams’ ability to timely recognize ADEs in this vulnerable patient population are limited to the Emergency Department setting [23].

Therefore, we conducted a multicenter cohort study with the aim to gain a detailed insight into recognition of ADEs by medical teams in older inpatients. In order to do so, both ADEs present upon admission and those occurring during the hospital stay were included in our analysis.

## Material and methods

### Study setting

The study was performed in the Internal Medicine wards of three teaching hospitals in the Netherlands: the Academic Medical Center (AMC) in Amsterdam, a 1,002-bed academic hospital, the Westfriesgasthuis (WFG) in Hoorn, a 506-bed regional teaching hospital, and the Spaarne Hospital (SH) in Hoofddorp, a 520-bed tertiary teaching hospital. The Internal Medicine wards were staffed by teams consisting of attending physicians, junior and senior residents, and interns on rotation, caring together for an average of ten adult patients daily. All three Hospital Pharmacy Departments offered only off-ward, daily clinical services, including preparation of parenteral medications by pharmacy technicians, on-call availability of a hospital pharmacist for pharmacotherapeutic or toxicological consultations, and therapeutic drug monitoring (TDM). In addition, Computerized Physician Order Entry (CPOE) systems with limited clinical decision support were in place in all three participating hospitals. These clinical decision support systems (CDSSs) generated three types of alerts based only on the prescribing data: drug–drug interactions, drug–drug duplications, and overdoses. No other sources, such as laboratory values or diagnostic tests results, were linked to these operating CDSSs.

### Study design

Data presented in this study are baseline results of an interrupted time-series study on the effects of the Ward-oriented pharmacy In Newly admitted Geriatric Seniors (WINGS) study [24]. Over a period of 8 months (April–November 2007), 250 patients aged 65 years or older were included in the study during three sampling periods: 90, 80, and 80 consecutively admitted patients were enrolled during the first (April–May 2007), second (July–August 2007) and third (October–November 2007) sampling period, respectively.

The WINGS study was conducted within the framework of the CAREFUL (pharmacist Coordinated ADE Reducing Efforts For Use in all Levels of healthcare) research program based on a cooperative effort between Leiden University Medical Center, AMC Amsterdam, University Medical Center Groningen, and University Medical Center Utrecht/Utrecht University.

### Patients

All patients aged 65 years or older who were taking five or more medications on the day of admission and who were admitted to an Internal Medicine ward of the participating hospitals during one of the three sampling periods were eligible for enrolment in the study. Patients were excluded if they were scheduled for chemotherapy, radiation therapy, or stem cell/kidney transplantation, were discharged within 24 h, and/or had been transferred from other hospitals or other non-medical wards within the study hospitals. Only the first hospitalization during the baseline period per each included patient was reviewed (index hospitalization). The index hospitalization included the day of admission and all days of the subsequent hospitalization until the patient was discharged home, transferred to a non-medical ward within the same hospital, or transferred to another healthcare facility (e.g., hospital, nursing home).

### Measurement and classification of ADEs

No golden standard currently exists for the measurement, definition, and assessment of ADEs [25, 26]. The definitions used in this study, as well as the method and assessment of ADEs follow recommendations by experts and are also widely accepted [4, 26–28]. An ADE was classified as any harmful event occurring during drug therapy that resulted either from appropriate care (non-preventable ADEs) or from medication errors (preventable ADE) [4]. Both errors of omission or commission were included in this study. Harmful events were defined as either abnormal laboratory values or clinical symptoms. ADEs were included if they resulted in an outbreak of new symptoms/pathology, in worsening of existing or new symptoms/pathology, or in a delay or lack of any expected improvement of existing or new symptoms/pathology [4].

A detailed description of the method applied in this study can be found elsewhere [24]. In summary, after an included patient was discharged or transferred, trained research nurses and pharmacy students first gathered all information available on the index hospitalization (medical and nurse patients’ files, medication charts, discharge letters and other medical correspondence, laboratory and diagnostic results) and completed a case report form (CRF) in which ‘so-called’ triggers were incorporated. These triggers were adapted from the Institute of Healthcare Improvement (IHI) ADE trigger-tool [29]. Examples of IHI ADE triggers are the ordering of vitamin K which may be related to overanticoagulation with coumarines, or a digoxin level of >2.0 μg/L which may be related to digoxin toxicity. These triggers were ticked off by the researchers when applicable, with an aim to prompt expert team attention to specific events which may potentially be ADEs [30]. Second, the information collected on the index hospitalization together with the CRFs were presented to two independent experts, namely, a senior specialist in Internal Medicine (LA) and a senior clinical pharmacist specializing in geriatric medicine (CS), both of whom were experienced in systematic ADE identification. These two experts reviewed all data independently and subsequently discussed their findings during scheduled meetings. A structured ADE assessment was used to determine the causality between a drug and an adverse event, preventability and severity of an ADE, recognition of ADEs by medical teams, and, when applicable, type of underlying medication error. Only ADEs with a causality score of nearly certain, probable, and possible, as assessed according to the World Health Organization–Uppsala Medical Centre (WHO-UMC) criteria were included [31]. ADEs caused by a medication error were classified as preventable. The severity of an ADE was scored according to Common Terminology Criteria for Adverse Events ver. 3.0 (CTCAEv3) developed by the U.S. National Cancer Institute [32]. For example, for hyperkalemia, severity of the ADE is classified as mild if the potassium concentration is higher than the upper limit of normal (5.5 mmol/L), as moderate if higher than 5.5–6.0 mmol/L, as severe if higher than 6.0–7.0 mmol/L, and as life-threatening if >7.0 mmol/L. Medication errors were classified according to the Dutch Central Medication Incidents Registration [33] and defined as errors in drug prescribing, transcribing, processing, compounding, stocking, dispensing, administering, or monitoring [33]. To assess if an medication error occurred, we utilized prevailing national [34] and local pharmacotherapeutic guidelines, as well as all alerts generated by the CDSSs operating in the participating hospitals.

The expert team assessed an ADE as unrecognized if, based on the patient charts reviewed, no indication was found that a medical team involved recognized patient harm as being medication related and/or no certain documented actions were targeted at that specific ADE.

During the scheduled meetings, the experts reached consensus on all aspects of the ADE assessment for an ADE to be included.

### Main outcome measures

The main outcome measures were: (1) rate of ADEs present upon admission and (2) rate of ADEs occurring during the hospital stay. Both outcomes were expressed as rates per 100 hospitalizations.

### Statistical methods

Descriptive statistics were applied for the analysis of patient characteristics, including means, standard deviations (SD), medians, and percentiles. To test for differences between patients included in the three hospitals, normally distributed continuous variables were compared using a one-way analysis of variance test and categorical variables were analyzed by using the chi-square test. Non-normally distributed continuous variables were compared using the Kruskall–Wallis test.

To compare the distributions of ADE severity, causality, type of harm manifestation, and type of events between unrecognized and recognized ADEs, we used the chi-square test or Fisher’s exact test.

Multivariable backward logistic regression analyses were conducted to determine which patient factors were independently associated with no recognition of ADEs present upon admission and which factors led to no recognition of ADEs occurring during hospitalization. Variables in the model for no recognition of ADEs present upon admission included age, sex, number of preadmission medications, the type of admission (elective or acute), the presence of cognitive impairment on admission (yes/no), and the Charlson Comorbidity Index score [35]. Variables in the model for no recognition of ADEs occurring during the hospital stay included age, sex, number of hospital medications, length of stay on an Internal Medicine ward, the presence of cognitive impairment on admission (yes/no), and the Charlson Comorbidity Index score [35]. A *p* value of <0.05 was considered to be statistically significant. Computer software SPSS ver. 18.0 (SPSS, Chicago, IL) was used for the calculations.

### Ethical considerations

The WINGS study protocol [24] was presented to The Medical Ethics Committee of the University of Amsterdam. The Medical Ethics Committee discussed the protocol and exempted it from review and official approval. According to the Dutch Medical Research Involving Human Subjects Act, such a review and approval were not required because the study did not involve direct interaction with human subjects. This research used retrospective patient chart review to assess the extent of suboptimal care related to ADEs. Therefore, the integrity of patients was not influenced, and all patient data were analyzed anonymously by coding each patient included in the study by a 6-digit number.

## Results

### Study population

Demographic characteristics of the 250 patients included in the study are shown in Table 1. The three groups of patients in the participating hospitals only differed in the median length of hospital stay on an Internal Medicine ward (median, with 25th and 75th percentile: AMC, 5.6 days (3.6, 7.9 days); WFG, 5.9 days (2.8, 8.1 days); SH, 7.4 days (4.8, 11.8 days); *p* = 0.025). Therefore, patients’ characteristics are in Table 1 presented after pooling.

**Table 1:** Patient characteristics

Characteristic	Total (*n* = 250)
Age, years (mean ± SD)	76.9 ± 7.5
Female, *n* (%)	133 (53.2)
Living independent, *n* (%)	211 (84.4)
Acute admission, *n* (%)	213 (85.2)
Length of stay, days, median (25th, 75th percentile)	5.9 (3.6, 9.6)
Specialty of wards, *n* (%)	
General internal medicine	98 (39.2)
Gastroenterology	51 (20.4)
Nephrology	46 (18.4)
Oncology and hematology	37 (14.8)
Rheumatology	18 (7.2)
Number of preadmission medications (mean ± SD)	7.3 ± 3.2
Number of hospital medications (mean ± SD)	11.0 ± 4.1
Number of concomitant diseases (mean ± SD)	3.16 ± 1.7
Most frequent types of diseases, *n* (%)	
Cardiovascular	179 (71.6)
Malignancy	94 (37.6)
Diabetes mellitus	84 (33.6)
Muscle skeletal	52 (20.8)
Renal	41 (16.4)
Pulmonary	39 (15.6)
Gastrointestinal	30 (12.0)
Neurologic	22 (8.8)
Psychologic	15 (6.0)
Charlson Comorbidity Index score, *n* (%)	
0 points	24 (9.6)
1–2 points	108 (43.2)
3–4 points	63 (25.2)
≥5 points	55 (22.0)
MDRD eGFR^b^ (ml/min/1.73 m^2^), *n* (%)	(*n* = 240)
≥90	21 (8.8)
60–89	73 (30.4)
29–59	88 (36.7)
15–28	34 (14.2)
<15	24 (10.0)

SD, Standard deviation; MDRD study, Modification of Diet in Renal Disease study; eGFR, estimated glomerular filtration rate

^a^Length of stay on the Internal Medicine wards

^b^For 10 patients no laboratory tests were run during the hospital stay to assess renal function

### Main outcomes

A total of 269 ADEs in 164 patients were identified. We found 60.4 ADEs (95 % CI 51.5–70.8) present upon admission per 100 hospitalizations (151 ADEs), of which 19.9 % (30/151) remained unrecognized during the subsequent hospitalization. During the hospital stay, we found 47.2 ADEs (95 % CI 39.4–56.5) per 100 hospitalizations (118 ADEs) to have occurred, of which 20.3 % (24/118) remained unrecognized (Table 2). When only severe, life-threatening, or fatal ADEs were considered, the overall proportion of unrecognized ADEs decreased from 20.1 to 7.8 %. When only ADEs assessed as having nearly certain or probable event–drug causality were considered, the overall proportion of unrecognized ADEs decreased from 20.1 to 15.2 %.

**Table 2:** Characteristics of adverse drug events identified and their in-hospital recognition by medical teams

Characteristics	Unrecognized ADEs (*n* = 54)	Recognized ADEs (*n* = 215)	*p* value and degrees of freedom
Time of identification			
ADE present upon admission	30 (55.6)	121 (56,3)	0.924; *df* = 1
ADE occurred during the hospital stay	24 (44.4)	94 (43,7)	
Severity			
Mild to moderate	33 (61.1)	109 (49.3)	0.171; *df* = 1
Severe or worse (life-threatening or fatal)	21 (38.9)	106 (50.7)	
Causality			
Nearly certain or probable/likely	41 (75.9)	191 (88.8)	0.014; *df* = 1
Possible	13 (24.1)	24 (11.2)	
Preventability			
Non-preventable	13 (24.1)	121 (56.3)	<0.001; *df* = 1
Preventable	41 (75.9)	94 (43.7)	
Type of events			
Clinical symptom	27 (50.0)	135 (62.8)	0.086; *df* = 1
Laboratory abnormality	27 (50.0)	80 (37.2)	
Type of harm manifestation			
New harm	9 (16.7)	174 (80.9)	<0.001; *df* = 1
Sustained/worsened harm or delayed recovery from harm	45 (83.3)	41 (19.1)	

ADE, Adverse drug event; df, degrees of freedom

Data are presented as the number of patients, with the percentage given in parenthesis

The expert team assessed 135 ADEs as preventable (50.2 %), i.e., caused by medication errors, of which the medical teams did not recognize 30.4 %. Medication errors most often identified were: omissions in prescribing (25.2 %), prescribing of too high or too low doses (25.2 %), prescribing of medication while contra-indicated (20.0 %), wrong pharmacotherapy choice for a known indication (excluding economic considerations) (11.1 %), drug–drug interactions (combinations should have been avoided or dosages adjusted) (7.4 %), drug administration errors (5.9 %), and lack of TDM (5.2 %).

The intra-rater agreement of the involved expert team (initial vs. second review by LA and CS 1 year later) was substantial for presence of an ADE (κ = 0.74), preventability of ADEs (κ = 0.68), and severity (κ = 0.93).

### Characteristics of recognized and unrecognized ADEs

A substantial number of ADEs identified by the expert team caused serious patient harm (47.2 %; 127/269 ADEs were scored as severe, life-threatening, or fatal). Of these ADEs 16.5 % were not recognized by the medical teams (Table 2). We identified four fatal ADEs (all recognized by the medical teams) and 38 ADEs which caused life-threatening patient harm [5 (15.2 %) unrecognized by the medical teams]. Differences in distributions between the unrecognized and recognized ADEs were found for causality (*p* = 0.014, *df* = 1), preventability (*p* < 0.001, *df* = 1), and type of harm due to an ADE (*p* < 0.001, *df* = 1). In comparison with recognized ADEs, unrecognized ADEs were more often ADEs with a possible drug–event causality score, preventable ADEs, and less often ADEs which resulted in new symptoms or pathology. The most frequent events related to ADEs (87.0 % of all ADEs identified), their severity and causality, and recognition are shown in Table 3, as well as medications most frequently involved in those ADEs listed per event type.

**Table 3:** Most frequently identified ADEs and their recognition by the medical teams during the hospital stay

Type of events (examples of most often involved medication)	No. of all events identified by the expert team (% unrecognized)	No. of severe or worse events (% unrecognized)	No. of nearly certain and probable events (% unrecognized)
Electrolyte disturbances (diuretics/RAAS inhibitors)	43 (18.6)	11 (27.3)	34 (17.6)
Hemorrhage (coumarines/anti-platelet medication, omissions of gastro-protective medication)	23 (0.0)	13 (0.0)	19 (0.0)
Central nervous system events^a^ (opiates/benzodiazepines/beta-blockers)	21 (23.8)	14 (21.4)	14 (7.1)
Hypotension/bradycardia (beta-blockers/diuretics/digoxin)	18 (27.8)	8 (12.5)	17 (29.4)
Delayed recovery from an infection or sustained infections^b^ (antibiotics)	18 (11.1)	15 (13.3)	16 (6.3)
Raised creatinine/renal insufficiency (antibiotics/NSAIDs/RAAS inhibitors/diuretics)	17 (47.1)	6 (33.3)	15 (53.3)
Constipation or ileus (omission of laxatives while taking opiates)	16 (6.3)	6 (0.0)	16 (6.3)
Hyper- and hypoglycemia (anti-diabetic drugs/corticosteroids)	15 (6.7)	10 (0.0)	14 (7.1)
Raised LTs/liver insufficiency (anti-diabetic drugs/antibiotics/statins)	15 (53.3)	7 (85.7)	10 (60.0)
Anemia^c^ (omission of iron supplements)	15 (40.0)	4 (25.0)	13 (38.5)
Raised INR (coumarines)	14 (0.0)	9 (0.0)	14 (0.0)
Skin reactions (intra-venous antibiotics)	10 (0.0)	0 (0.0)	10 (0.0)
Nausea and vomiting (antibiotics)	9 (44.4)	3 (33.3)	7 (28.6)

RAAS, Renin–angiotensin–aldosterone system; NSAIDs, non-steroidal anti-inflammatory drugs; LTs, liver transaminases; INR, International Normalization Ratio

^a^Mainly delirium (7 ADEs), extrapyramidal symptoms (4 ADEs), falls (4 ADEs), and somnolence/drowsiness (3 ADEs)

^b^Delayed recovery from or sustained infections were primarily caused by inappropriate empirical antibiotic therapy choice, too short treatment regimes, or inappropriate route of antibiotic administration (oral where intravenous was indicated)

^c^Mainly cases of older patients with chronic cardiovascular disease who were hospitalized due to (excessive) blood loss, in whom anemia was not sufficiently corrected to decrease risks involved with low hemoglobin values

All ADEs resulting in hemorrhage, raised International Normalization Ratio (INR), skin reaction, and, except for one event per category, all ADEs resulting in constipation or ileus, and hyper- and hypoglycemia were recognized by the medical teams involved (Table 3). Causality of those events with medication was assessed as nearly certain or probable for 64.3 % of the ADEs resulting in raised INR to 100.0 % of the skin reaction ADEs and ADEs resulting in constipation/ileus. More than half of ADEs resulting in hemorrhage (56.5 %) and hyper-hypoglycemia (66.7 %), and all ADEs resulting in raised INR (100.0 %) were severe or worse according to the CTCAEv3 criteria [32],

The proportions of unrecognized ADEs were higher for events in the following categories: central nervous system (CNS), (23.8 % unrecognized), hypotension/bradycardia (27.8 % unrecognized), anemia (40.0 % unrecognized), nausea and vomiting (44.4 % unrecognized), raised creatinine/renal insufficiency (47.1 % unrecognized), and raised liver transaminases (LTs)/liver insufficiency (53.3 % unrecognized) (Table 3). Of the latter two categories, 75.5 % manifested as an abnormal laboratory test result only. The majority of these ADEs was assessed as having nearly certain or probable drug causality (66.7 % for CNS events to 94.4 % for hypotension/bradycardia). The proportions of severe or worse ADEs according to CTCAEv3 criteria [32] were, except for CNS events, lower (26.7 % for anemia to 44.4 % for hypotension/bradycardia) than those for ADEs which were (almost) all recognized. Of the CNS events, 66.7 % was scored as severe or worse.

### Multivariable analyses

The Charlson Comorbidity Index score [32] was the only characteristic independently associated with no recognition of ADEs present upon admission [odds ratio (OR) 0.76, 95 % CI, 0.59–0.97; *p* = 0.026). Patients with cognitive impairment on admission seemed to have a higher risk for unrecognized ADEs being present upon admission in comparison to patients without cognitive impairment (OR 2.4, 95 % CI 1.00–5.85; *p* = 0.05). No independently associated patient characteristics were found for having an unrecognized ADE occurring during the hospital stay.

## Discussion

Our study focused on medical teams’ ability to recognize ADEs in hospitalized older patients aged 65 years or older and included an assessment of both ADEs present upon admission and ADEs occurring during the hospital stay. We found that of all 269 ADEs identified by our expert team, 20 % remained unrecognized by medical teams during the hospital stay. Of the unrecognized ADEs, more than a half (56 %) were ADEs present upon admission, and the majority (76 %) were ADEs caused by medication errors. Our results show that patients’ characteristics as well as ADE characteristics impact the ability of medical teams to recognize ADEs in older inpatients.

The rates of both ADEs present upon admission (60.4 per 100 hospitalizations) and ADEs occurring during the hospital stay (47.2 per 100 hospitalizations) identified in this study are markedly higher than those reported in previous ADE studies in older patients [5, 12–22]. These differences can partly be explained by differences in the type of identification method used, the clinical setting, and in the definitions of the outcomes applied [25, 36, 37]. In this study, we used a definition of an ADE which included not only new ADEs, but also worsened and sustained harm or delayed recovery from harm due to both preventable and non-preventable ADEs [4, 24]. Furthermore, by utilizing an adapted IHI ADE trigger-tool [29] as an aid, our chart review was more structured and may, therefore, be more accurate [27, 30, 38]. We also deployed a physician–pharmacist team to review patient charts because the professional knowledge of this combination is complementary [39]. These combined aspects of our methodology may have resulted in a higher number of ADEs being identified. In addition, we included patients with five or more medications on the day of admission. It is well acknowledged that a higher number of medications significantly increases the risk of an ADE [40]. The distribution of ADEs in our study per severity category, however, is comparable to that in other published ADE studies reporting on severity classification [23, 41], indicating that the high ADE yield gained in this study was, therefore, also not merely a result of a higher identification of less clinically relevant ADEs.

Although a direct comparison of our results on ADE recognition in older medical inpatients with those of previous studies is not possible, some aspects of our findings can be compared. In a study by Hohl et al. [23] conducted in an Emergency Department (ED) setting, the medical teams did not recognize 50 % of ADEs present upon admission in older patients, whereas in our study this proportion was 20 %. This lower proportion can be explained by several factors. First, the ED study of Holhl et al. [23] was conducted in a Canadian hospital [23] and our study was conducted in three hospitals in the Netherlands. As such, the level of medical training in geriatrics and/or ADEs awareness may differ between the physicians involved in these two studies. Differences between healthcare settings are known to have an impact on ADE rates [36]. Second, in comparison to the relatively short ED visit, the time available to critically review admission medication during the hospital stay is longer. Third, because more than 80 % of our patients were acutely admitted, they were often first seen in the ED. Hospital admission summaries written by ED physician’s sometimes included possible medication-related causes promoting further examination by the treating physicians on medical wards. Interestingly, the percentage of unrecognized ADEs in our study is in line with that reported in a study in a general inpatient population where 24 % of ADEs were not recognized by the medical team [11]. Given the complexity of older patients’ cases, one could expect the proportion of unrecognized ADEs in our study to be much higher. Unfortunately, detailed data on the type of unrecognized ADEs were not presented by Nebeker et al. [11], which hampers further exploration of this point.

### Patient characteristics and ADEs recognition

The results of our multivariate analyses showed that the odds for having an unrecognized ADE on admission decreased by 24 % with each one point increase in the Charlson Comorbidity Index score [35] (95 % CI 0.59–0.97; *p* = 0.026), while these odds appeared to be 2.4-fold higher in patients with cognitive impairment (95 % CI 1.00–5.85; *p* = 0.05). The former results indicate that the physicians were well aware of the frail state of multi-morbid patients; the latter results suggest, however, that they were insufficiently aware of medication-related harm in patients with cognitive impairment on admission. A possible explanation for the better recognition of ADEs in multi-morbid patients is probably the investment of more time and/or involvement of other medical specialties. Previous studies have shown that a multidisciplinary approach is successful in improving prescribing in older patients [42–44]. Cognitive impairment is a well-known and highly prevalent atypical symptom in hospitalized elderly patients [45]. Atypical disease presentation often hampers correct and timely diagnosis and treatment [9]. Therefore, such presentation may be an extra barrier in distinguishing between disease and medications as a cause of patients’ symptoms that are present at the time of admission.

### Characteristics and recognition of ADEs

In our study, recognized ADEs were more often ADEs manifesting as new symptoms (*p* < 0.001; *df* = 1). This finding is in line with the results reported by Hohl et al. [23] who found that ED physicians were most skillful in recognizing ADEs that represented patients’ chief complaints. In a daily practice on the wards, physicians tend to focus first on new symptoms when making differential diagnoses. The most plausible causes are listed, and actions prioritized according to urgency and/or severity of the symptoms. Worsening of existing complains, delayed recovery, or no clinical improvement are probably more often associated with progression of the underlying pathology or the frail state of an older patient than with a drug effect.

As already mentioned, the task of distinguishing between an ADE and other causes of an event in often multi-morbid, polymedicated older inpatients is challenging [6, 9]. Our results show that a strong causality between an event and a drug (nearly certain or probable ADEs) improves the ability of a physician to recognize ADEs (*p* = 0.014, *df* =1). The essential distinctions between nearly certain/probable and possible drug–event causality are that in the latter case there may be another equally likely explanation for the event and/or there is no information or uncertainty regarding what has happened after the suspected medication was stopped [31]. Therefore, it is likely to assume that possible ADEs are more easily missed. Not recognizing possible ADEs can, however, have serious consequences, as illustrated by one of our cases—that of a 90-year-old man, recently started on mirtazapine 15 mg once daily for depression, presented with dyspnoea, peripheral edema, and somnolence. The reported incidence of somnolence with mirtazapine use is >1–10 % and of peripheral edema >10 %. The patient was, however, diagnosed with pneumonia, and antibiotic treatment was initiated. The peripheral edema was treated with intravenous furosemide boluses; treatment with mirtazapine was continued. One day post-discharge, the patient was readmitted with increased somnolence and peripheral edema. After consulting a geriatrician, mirtazapine was discontinued, with subsequent resolution of the somnolence and peripheral edema.

In our study, CNS events, such as somnolence or delirium, as well as hypotension/bradycardia, anemia, nausea/vomiting, raised creatinine/renal insufficiency, and raised LTs/liver insufficiency were often unrecognized as being drug-related (>20 % unrecognized). These less well-recognized events are examples of symptoms mimicking the presentation of various underlying pathologies and are less specific side-effects of medications. Moreover, in our study, these types of ADEs represented 40 % of the most frequently identified ADEs (Table 3) and have also been reported as frequent events in other ADE studies in older inpatients [5, 12–22]. It would appear that sufficient pharmacotherapeutic and geriatric knowledge is necessary to be able to identify these evidently more challenging ADEs [43, 44]. In the hospitals participating in this study, the day-to-day care of inpatients is, however, provided by mostly junior medical residents with 1 or 2 years of clinical experience. Studies on the level of geriatric competencies of medical residents show that there are gaps in their skills and knowledge that need to be addressed to ensure that the growing group of older inpatients receive safe care [46, 47]. In case of ADEs resulting in raised creatinine/renal insufficiency and raised LTs/liver insufficiency, the fact that majority of these resulted in abnormal laboratory values only (76 %) may additionally have attributed to the lower recognition of these events (*p* = 0.086, *df* = 1). In the study by Hohl et al. [23], the ER physicians were also less proficient at detecting ADEs which resulted in abnormal laboratory values, i.e., the so-called “silent ADEs”.

In contrast, ADEs resulting in hemorrhage, raised INR, constipation/ileus, skin reactions, and hyper/hypoglycemia were (almost) all recognized as being drug-related. These ADEs are examples of events with clinically apparent consequences and are very common and specific side-effects of medications. For example, the INR is often closely monitored during the hospital stay in patients taking coumarines. According to CTCAEv3 criteria [32], a severely raised INR implies an INR twofold the upper limit of normal (ULN) (9/14 cases of raised INR in our study). For atrial fibrillation, the INR target range is 2.5 to 3.5, and twofold the ULN indicates an INR of >7.0. This clinically relevant rise, a known effect and side-effect of coumarines, was, therefore, easily noticed (100 % recognized). The fact that the majority of these well-recognized ADEs caused severe or worse patient harm (57–100 %) may also have contributed to better recognition. The unrecognized ADEs seem more likely to be ADEs of mild or moderate severity (*p* = 0.171, *df* =1).

Last but not least, in our study the majority of ADEs (70 %) occurring during the hospital stay were caused by medication errors. Prescribing contra-indicated medications, dosing errors, and drug omissions accounted for 70 % of all errors identified. Moreover, unrecognized ADEs were significantly more often preventable ADEs (*p* < 0.001). Many medications used by older patients are lifelong treatments, often prescribed (previously) by other medical specialists or general practitioners for known therapy needs and conditions at that time [6]. However, these conditions and needs can change, and medication once chosen could become inappropriate [6]. Physicians’ reluctance to change or question drug therapies prescribed by colleagues is a well-known phenomenon [48] and a possible reason why (home) medication, even with errors, was continued unjustifiably.

### Limitations

Our study has a number of limitations. First, methods based on expert opinion in the identification of ADEs are known to have a low agreement between the experts involved [49]. Given the obvious differences in knowledge and expertise between pharmacists and physicians, such a disagreement is, however, expected [50]. Yet, exactly because of these differences, pharmacists and physicians are complementary experts in terms of ADE identification [39]. Differences between our two experts regarding their judgments on ADE causality, severity, preventability, and recognition were resolved by consensus. Moreover, the identification and assessment of ADEs by our expert team were reproducible. Second, because we identified ADEs based on a retrospective patient chart review, a registration bias may have occurred. Although a prospective ADE identification method has been shown, especially for preventable ADEs, to provide more veracious results [51], an unresolved dilemma remains because the prospective method can also bias results given physicians’ awareness of data collection. To use the data presented in this manuscript for an evaluation of future interventions, we determined that a retrospective method was more suitable to our needs. Third, a patient chart review method is especially useful to identify preventable ADEs due to prescribing errors and less helpful to identify preventable ADEs due to administration errors [52]. This may explain why administration errors accounted for only 6 % of the preventable ADEs identified in this study. We were, however, mainly interested in the suboptimal care from the medical perspective and less from the nursing perspective. Although administration errors are frequent, the majority of these errors do not lead to ADEs [3]. Finally, the measurement of quality of care based on chart reviews is prone for documentation bias [53]. Actions taken and considerations regarding the choice of therapy may not be evident from what was recorded in patient charts and could, therefore, be considered inappropriate. However, it is also well-known that actions documented in patients files are not always performed [53]. Therefore, we collected among other things medical files, nursing files, medication charts, medical correspondence, laboratory and diagnostic findings, discharge letters, and home and discharge medication lists. Our experts considered all of these different types of sources during the structured assessment of the ADEs. Consequently, we are confident that our chart review provides veracious results for both ADEs rates and their recognition by medical teams [54].

### Unanswered questions

Medication errors often have a complex causality and arise not only from active errors, such as insufficient knowledge, but also from factors such as a lack of training in prescribing or insufficient supervisor’s feedback on prescribing [55]. The opportunity to investigate these so-called error provoking and latent conditions was limited because we identified ADEs by a retrospective patient chart review. By involving the Internal Medicine staff and residents in a risk-analysis to design future interventions, we hope to gain more insight into these factors [56].

### Future research

Our data were obtained in three different hospitals, which increases the generalizability of our results. However, because the degree of ADEs recognition may differ between medical specialties or countries [11, 23], more studies are needed to confirm our findings. Strategies such as the Screening Tool of Older Persons’ potentially inappropriate Prescriptions (STOPP), Screening Tool to Alert doctors to the Right Treatment (STOP) [57], and quality indicators for in-hospital pharmaceutical care of elderly patients [58] have the potential to improve the recognition of ADEs by detecting inappropriate prescribing. Considering ADEs as a differential diagnosis in older patients should be a standard approach on admission and during the hospital stay. Medical education regarding ADEs, a regular and comprehensive medication review [6, 10, 59], a daily participation of clinical pharmacists in medical teams on the wards [60–62], and CDSSs [63, 64] specific to older inpatients’ medication risks [65, 66] could all be of an added value in reducing preventable ADEs and improving ADE recognition.

## Conclusions

By applying a comprehensive measurement strategy that included the identification of ADEs present upon admission and those occurring during the hospital stay, followed by an assessment of medical teams’ ability to recognize these ADEs, we were able to identify patient- and ADE-related factors influencing the ability of medical teams to recognize ADEs in older vulnerable patients. The medical teams involved performed best at recognizing ADEs manifesting as new or clinically apparent symptoms and those causing serious patient harm. Less specific ADEs mimicking underlying pathology, ADEs with only an abnormal laboratory value or mild to moderately severe ADEs were less well recognized. However, physicians should also aim at achieving timely recognition of such less critical and less evident ADEs to prevent future emergencies. The findings of this study suggest areas where physicians should focus their attention in order to further improve their ability to recognize ADEs.
